# Yoga Modulates Alzheimer's Disease Pathophysiology by Targeting Key Biomarkers, Oxidative Stress, Inflammation, and Neurocognition

**DOI:** 10.1002/alz70856_097301

**Published:** 2025-12-24

**Authors:** Meenakshi Kaushik, Manjari Tripathi, Rima Dada

**Affiliations:** ^1^ All India Institute of Medical Sciences, New Delhi, Delhi, Delhi, India; ^2^ All India Institute of Medical Sciences, New Delhi, Delhi, India

## Abstract

**Background:**

Alzheimer's disease (AD) is characterized by cognitive decline, neuroinflammation, and synaptic dysfunction. This study explores the impact of a 12‐week yoga intervention on cognitive function, mood, biomarkers, inflammatory responses, and oxidative stress in AD patients.

**Method:**

Out of 60 patients initially enrolled, 50% were lost to follow‐up, resulting in the evaluation of 30 AD patients (mean age 66.4 ± 3.80 years) and 20 healthy controls (HC; mean age 64.4 ± 2.40 years). Cognitive function was assessed using the Montreal Cognitive Assessment (MoCA) and Geriatric Depression Scale (GDS). AD biomarkers, including amyloid‐beta (Aβ), pTau proteins (pTau 181 and pTau 217), APOE, APOE4, and inflammatory markers (IL‐6, IL‐11, IL‐15, IL‐37, TNF‐α, TGF‐β1, CRP, BDNF), were measured. Oxidative stress was assessed by 8‐hydroxy‐2‐deoxyguanosine (8‐OHdG). Further analyses included ROC curves and Pearson correlations between biomarkers and cognitive scales.

**Result:**

The AD group showed significant cognitive improvement post‐intervention, with MoCA scores increasing and GDS scores decreasing (*p* < 0.01). Cognitive domains such as language, attention, and memory were enhanced. Biomarker analysis revealed reductions in Aβ‐40 (*p* < 0.05), pTau 181, pTau 217, CRP, APOE, and 8‐OHdG, while Aβ‐42 (*p* < 0.05), Aβ‐42/Aβ‐40 ratio (*p* < 0.05), and BDNF increased (*p* < 0.05). Inflammatory markers IL‐6, TNF‐α, IL11, IL15, IL37, and IL‐11 decreased, while TGF‐β1 increased (*p* < 0.05). Correlations revealed cognitive improvement (MoCA) positively associated with Aβ‐42 (*r* = 0.65, *p* < 0.01) and inversely with IL‐6 (*r* = ‐0.58, *p* < 0.05). Mood improvements (GDS) were inversely correlated with APOE levels (*r* = ‐0.62, *p* < 0.01).

**Conclusion:**

Yoga intervention significantly enhanced cognition, mood, and biomarker profiles, including reductions in inflammatory markers, APOE, and oxidative stress, with positive correlations between cognitive improvement and Aβ‐42, and inverse associations with IL‐6. These findings support yoga as a promising therapeutic strategy in AD.

